# White matter tracts contribute selectively to cognitive functioning in patients with glioma

**DOI:** 10.3389/fonc.2023.1221753

**Published:** 2023-10-20

**Authors:** Mia Andreoli, Melissa-Ann Mackie, David Aaby, Matthew C. Tate

**Affiliations:** ^1^ Feinberg School of Medicine, Northwestern University, Chicago, IL, United States; ^2^ Department of Neuropsychology, Northwestern Memorial Hospital, Chicago, IL, United States; ^3^ Department of Neurological Surgery and Neurology, Northwestern Memorial Hospital, Chicago, IL, United States

**Keywords:** glioma, white matter tracts, diffusion tensor imaging, fractional anisotropy, cognitive function, neuropsychology, surgical planning

## Abstract

**Objective:**

The functional organization of white matter (WM) tracts is not well characterized, especially in patients with intrinsic brain tumors where complex patterns of tissue injury, compression, and neuroplasticity may be present. This study uses diffusion tensor imaging (DTI) to investigate the relationships between WM tract disruption and cognitive deficits in glioma patients.

**Methods:**

Seventy-nine patients with glioma underwent preoperative DTI and neuropsychological testing. Thirteen WM tracts were reconstructed bilaterally. Fractional anisotropy and streamline number were obtained for each tract as indices of connectivity. Univariate regression models were used to model the association between WM tract connectivity and neuropsychological outcomes.

**Results:**

Glioma patients exhibited variable injury to WM tracts and variable cognitive deficits on validated neuropsychological tests. We identified 16 age-adjusted associations between WM tract integrity and neuropsychological function. The left inferior frontal-occipital fasciculus (IFOF) predicted list learning and dominant-hand fine motor dexterity. The right IFOF predicted non-dominant-hand fine motor dexterity and visuospatial index scores. The left inferior longitudinal fasciculus (ILF) predicted immediate memory list learning and index scores. The right ILF predicted non-dominant-hand fine motor dexterity and backward digit span scores. The left superior longitudinal fasciculus (SLF) I predicted processing speed. The left SLF III predicted list learning, immediate memory index scores, phonemic fluency, and verbal abstract reasoning. The left cingulum predicted processing speed. The right anterior AF predicted verbal abstract reasoning.

**Conclusion:**

WM tract disruption predicts cognitive dysfunction in glioma patients. By improving knowledge of WM tract organization, this analysis may guide maximum surgical resection and functional preservation in glioma patients.

## 1 Introduction

White matter (WM) tracts serve a crucial role in the structural basis of higher cognition, including attention, memory, language, visuospatial skills, and executive functions (1, 2). WM mediates the efficient transfer of information across distributed neural networks of myelinated axons, and lesions to WM tracts produce variable disconnection syndromes resulting from local and widespread disruption of brain connectivity (3). There is significant interindividual variability in WM, especially when considering brain pathologies (4). Thus, mapping WM phenotypes may reveal important differences in structure and function, leading to more individualized care (4). Nonetheless, the functions of specific WM tracts are not well characterized, especially in glioma patients, where complex patterns of WM tract infiltration, compression, and neuroplasticity may be present (1, 5, 6). These patients often face a poor prognosis and neurological deficits resulting from the tumor and from treatment, which negatively impact quality of life (7, 8). Therefore, a better understanding of the functional significance of WM tracts is essential to optimize tumor resection, radiation planning, and overall cognitive preservation.

The ability to non-invasively study WM tracts has advanced in recent years due to diffusion tensor imaging (DTI), an MRI-based technique that measures the translational motion of water (9). Tractography is a DTI modeling technique that enables preoperative visualization of WM tracts in relation to a tumor (10, 11). It has been adapted by neurosurgeons to assist in surgical planning and to facilitate safe resection of a tumor while minimizing the risk of damage to critical neural fibers (11). DTI is also used in quantitative studies through the extraction of microstructural parameters like fractional anisotropy (FA), which indicates the direction of diffusion of water along a WM tract and is the most commonly used biomarker for tract integrity (1, 4, 9). A high FA value indicates organized directional diffusion of water, and thus, greater fiber integrity (12). A reduction in FA values in the perilesional zone around gliomas is suggestive of a loss of tract integrity characterized by both axonal disorganization and increased cellular water (6). Volumetric parameters like the number of streamlines in a WM tract can also be used as a measure of WM connectivity, representing axonal damage or degeneration (4).

DTI has been used extensively to examine the relationship between WM tracts and cognition in a number of common neurological diseases, such as stroke, but less commonly for brain tumors (4, 9, 10). Compared to other etiologies, gliomas typically exhibit slower growth kinetics and manifest as relatively focal lesions (13). They are associated with variable cognitive dysfunction, which is generally mild, particularly in the case of low-grade gliomas, due to the brain’s neuroplastic capacity to compensate over time (13, 14). Therefore, specific neuropsychological deficits may indicate damage to a functionally critical structure (13). Previous studies have shown that gliomas alter WM tract integrity due to their infiltrative growth and preferential invasion along WM tracts (15), which may disrupt networks related to cognitive function (5, 16, 17). Incekara et al. investigated three WM tracts and cognitive domains in glioma patients, and discovered a connection of the arcuate fasciculus (AF) and inferior fronto-occipital fasciculus (IFOF) with language and attention (16). Kinoshita et al. demonstrated that increased postoperative FA of the AF was associated with improved language scores in glioma patients (17). Liu et al. reported a correlation between visuospatial assessments and the temporal part of the right superior longitudinal fasciculus (SLF) in 35 glioma patients using whole-brain tractography analysis (5).

In this study, we analyzed DTI data collected from 79 glioma patients to investigate how gliomas impact the structural integrity of 13 major WM association tracts. In addition, we assessed the relationship between WM structural alterations and performance on a wide range of neuropsychological tests. This analysis may provide valuable information for neuroscientists to better understand the mechanisms of functional organization of the human brain. Additionally, it may be used to optimize individualized treatment planning and counseling for patients with brain tumors.

## 2 Methods

### 2.1 Participants

This study included 79 patients with glioma who underwent preoperative DTI and neuropsychological testing between August 2015 and December 2020 (**Table 1**). Approval of the research protocol was obtained from the Institutional Review Board and informed consent was obtained from each participant. The mean age of participants was 47 (range, 19–76) years. Forty-two (53%) were men, 36 (46%) were women, and 1 (1%) was transgender. The majority (89%) were right-handed. Gliomas were located throughout the brain in the right (46%), left (46%), or bilateral (9%) hemispheres. Gliomas spanned the frontal (56%), temporal (34%), parietal (28%), and occipital (2%) lobes and the insula (9%), or were multifocal (5%). All tumor grades were represented and there was an even mix of isocitrate dehydrogenase (IDH) wild-type (39%) and IDH mutant (38%) tumors, with information about IDH mutation status not available for 18 participants (23%). To provide additional context, clinical DTI reports were reviewed for each patient and the frequency of reported tract involvement was recorded.

**Table 1:** Clinical and demographic data of patients with glioma in this study.

Characteristic (*N* = 79)	Mean (SD) [Minimum–Maximum]; *n* (%)
Age	47 (16) [19–76]
Gender	
Male	42 (53%)
Female	36 (46%)
Transgender	1 (1%)
Handedness	
Right	70 (89%)
Left	7 (9%)
Ambidextrous	2 (2%)
Brain tumor grade (WHO)	
1	2 (2%)
2	18 (23%)
3	23 (29%)
4	36 (46%)
Lesion hemisphere	
Right	36 (46%)
Left	36 (46%)
Bilateral	7 (9.0%)
Lesion lobe*	
Frontal	44 (56%)
Temporal	27 (34%)
Parietal	22 (28%)
Occipital	2 (2%)
Insula	7 (9%)
Multifocal	4 (5.%)
IDH	
Wild-type	31 (39%)
Mutant	30 (38%)
None available	18 (23%)

*Percentages do not add up to 100% because some participants had a lesion involving more than one lobe.

### 2.2 Neuropsychological testing

All patients were evaluated preoperatively with a neuropsychological protocol that included testing of memory, language, attention, visuospatial, and executive functions. Their baseline level of cognitive ability was estimated using the Test of Premorbid Functioning (TOPF) (18).

Attention was assessed using tests from the Repeatable Battery for the Assessment of Neuropsychological Status [RBANS (19);] and the Wechsler Adult Intelligence Scale – Fourth Edition (WAIS-IV) (20). These tests of attention included RBANS coding, RBANS digit span, WAIS-IV forward digit span, and WAIS-IV backward digit span. Visuospatial ability was assessed with the RBANS figure copy and line orientation tasks. Immediate memory was assessed with the RBANS list learning and story memory tasks. Delayed memory was assessed with the RBANS list recognition, list recall, story recall, and figure recall tasks.

Language was assessed with visual naming, semantic fluency, and phonemic fluency tasks. Visual naming tasks included the RBANS 10-item test and the 31-item Neuropsychological Assessment Battery (NAB) Naming Test. Semantic fluency was assessed using two separate tests requiring participants to name fruits and vegetables (19) or animals (21). Phonemic fluency was performed with three trials of producing words beginning with F, A, and S, respectively, in 1 min (21).

Executive functions were assessed using the Stroop Color–Word Test to assess cognitive interference, the Trail Making Test Parts A & B (22) to assess executive function and processing speed, and the WAIS-IV similarities test (20) to assess verbal abstract reasoning. Fine motor dexterity was assessed with the Grooved Pegboard Test, with separate trials for the dominant hand (DH) and non-dominant hand (NDH). Social cognition was sampled using an affect recognition task (23). Current mood symptoms were assessed with the self-reported Beck Anxiety and Depression Inventories.

Additionally, six age-corrected RBANS index scores, transformed to *z*-scores relative to the normative sample, were calculated for a total score, as well as scores for immediate memory, delayed memory, visuospatial, attention, and language.

### 2.3 Image acquisition and processing

As part of a standard preoperative imaging protocol, participants were scanned using a 3T MRI imaging system, with intravenous gadolinium contrast. T1 images were obtained with 192 slices at a thickness of 1.0 mm. Diffusion sequences were obtained in 64 independent directions, with a slice thickness of 5.2 mm and a *b* value of 1,000 s/mm^2^. DTI acquisition was used to generate directional color FA maps. These were registered to individual anatomy and reformatted in three planes. DTI and 1-mm T1 images were fused using the automated Brainlab Elements® Image Fusion software.

### 2.4 Tractography overview

**Figure 1:**
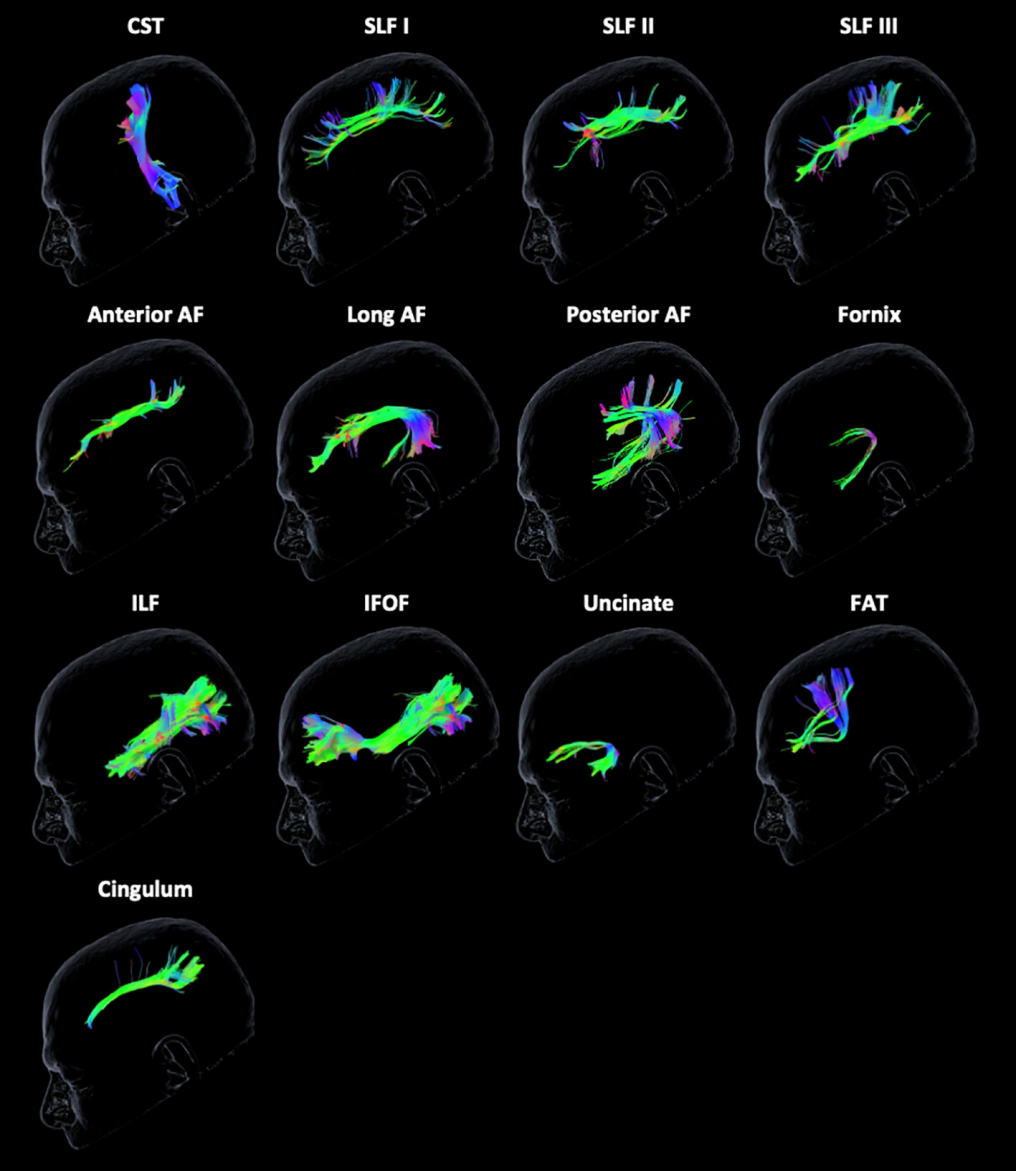
Tractography reconstruction of white matter tracts. The following tracts are displayed from left to right from the unaffected contralateral hemisphere of a glioma patient: corticospinal tract (CST), superior longitudinal fasciculus (SLF) I/II/III, anterior, long, and posterior segments of the arcuate fasciculus (AF), fornix, inferior longitudinal fasciculus (ILF), inferior frontal-occipital fasciculus (IFOF), uncinate fasciculus, frontal aslant tract (FAT), and cingulum. The color scheme represents the orientation of the major eigenvector: blue is superior to inferior, red is left to right, and green is anterior to posterior.

The Brainlab Elements® deterministic dual-tensor tracking algorithm was used to reconstruct 13 WM tracts bilaterally, including the anterior, long, and posterior segments of the arcuate fasciculus (AF), the superior longitudinal fasciculus (SLF) I/II/III, the corticospinal tract (CST), the frontal aslant tract (FAT), the uncinate fasciculus (UF), the inferior longitudinal fasciculus (ILF), the inferior fronto-occipital fasciculus (IFOF), the fornix, and the cingulum (**Figure 1**). These tracts were selected because they are relatively large, frequently studied fiber bundles with possible functional relevance and previously published guidelines for tract reconstruction (2, 24). The researcher performing tractography was blinded to neuropsychological scores at the time of tract reconstruction. By examining each WM tract bilaterally in all participants, we were able to compare the FA values of injured tracts to those of the unaffected contralesional tracts.

Tracking was initiated using default parameters for minimum streamline length (80 mm), maximum angulation (20°), and minimum FA (0.2) to reduce false-positive tracts. FA may be less than the 0.2 threshold in tracts infiltrated by a tumor, causing tracking to terminate prematurely. However, the algorithm requires constraints to avoid erroneous overestimation of streamlines (25). If there were zero streamlines present between multiple ROIs due to a large tumor volume causing extensive anatomical disruption (occurring in 6% of tracts), the FA value for that tract could not be obtained and was excluded from the analysis. Streamlines that were clearly extraneous or crossed to the opposite hemisphere were pruned manually.

### 2.5 White matter tract reconstruction

All regions of interest (ROIs) were based on guidelines established by experts in the field. The ROIs used to reconstruct the AF segments, SLF I/II/III, CST, FAT, cingulum, UF, ILF, and IFOF were based on the guidelines established by Rojkova et al. (2) and Catani et al. (24). The fornix was reconstructed based on the guidelines from Catani et al. in combination with additional previously published ROIs (24, 26, 27).

The AF is an association tract that connects the perisylvian language areas of the frontal, parietal, and temporal lobes. The AF has been characterized as comprising three segments: the anterior, long, and posterior segments (28, 29), which are utilized in the present study. However, it is important to note that the structure of the AF is still debated, and other authors describe a dorsal and ventral component of the AF with a spatially separate temporo-parietal aslant tract (30, 31). To isolate the AF tracts, three sagittal ROIs were delineated (1): an ROI around the ventral premotor, pars opercularis, and posterior portion of the middle frontal gyrus (i.e., Broca territory) (2), an ROI around the supramarginal gyrus and angular gyrus (i.e., Geschwind territory), and (3) an ROI around the posterior temporal lobe and Heschl gyrus (i.e., Wernicke territory). The anterior AF included ROIs #1 and #2, the long AF included ROIs #1 and #3, and the posterior AF included ROIs #2 and #3.

The SLF is an association bundle, composed of three segments, that connects the frontal and parietal lobe. These tracts were delineated using a coronal ROI around the parietal lobe at the level of the posterior commissure and a coronal ROI around either the superior, middle, or inferior frontal gyrus at the level of the anterior commissure to isolate SLF I, II, and III, respectively. An axial region of avoidance was included in the WM of the temporal lobe to exclude the temporo-frontal connections belonging to the arcuate fasciculus.

The CST is a vertically oriented bundle that projects from the precentral gyrus of the cerebral cortex to the spinal cord. The CST was isolated with an ROI around the precentral gyrus on a surface projection view and a second axial ROI around the ipsilateral cerebral peduncle.

The cingulum is a ring-shaped bundle that connects frontal, parietal, and medial temporal regions bordering the corpus collosum. The cingulum was isolated with a sagittal ROI delineated around the left and right WM of the cingulum. A sagittal ROI of the corpus callosum was excluded.

The FAT connects the supplementary motor cortex and lateral superior frontal gyrus with the inferior frontal gyrus. The FAT was isolated using two ROIs encompassing the superior and inferior frontal gyrus on a 3D surface projection view.

The IFOF, ILF, and UF are three large association bundles that connect the orbitofrontal cortex, anterior temporal lobe, and occipital lobe. Three bilateral ROIs were delineated to isolate these tracts (1): a coronal ROI around the external and extreme capsule (2), a coronal ROI containing the occipital lobe, and (3) an axial ROI containing the anterior temporal region. The IFOF included ROIs #1 and #2, the ILF included ROIs #2 and #3, and the UF included ROIs #1 and #3.

The fornix is a C-shaped tract found in the mesial aspect of the cerebral hemispheres. The fornix was isolated using three large rectangular ROIs, including two axial ROIs at the level of the columns and crus of the fornix and one coronal ROI intercepting the body of the fornix in the center of the thalamus.

### 2.6 Statistical analysis

All statistical analyses were conducted using R Statistical Software version 4.1.2. Descriptive statistics, including mean ± standard deviation and count (percentage), were used to report on demographic characteristics of the sample. Exploratory analyses were used to assess normality in the data on neuropsychological outcomes. Skewed neuropsychological outcomes were log-transformed. Outcomes that remained skewed after log transformation were modeled as Poisson variables rather than as continuous in all subsequent analyses.

We used univariate regression models to model the association between each neuropsychological outcome variable and each streamline number and FA predictor of interest, for a total of 1,768 models (i.e., 52 univariate models associated with each outcome variable). We also analyzed a second set of models, which were additionally adjusted for age because FA values and neuropsychological outcomes decrease linearly with age (32). The RBANS index scores were already adjusted for age and thus are excluded from these additional models (for a total of 1,457 models). The false discovery rate (FDR), or the expected proportion of discoveries that are falsely rejected, was used to correct for multiple testing. While still controlling for the proportion of type I errors at a predetermined alpha level, FDR has been shown to have greater power to detect true positives compared to more conservative methods such as the Bonferroni correction (33). We used a common FDR of 5%. Only models that remained significant after the FDR correction are reported. Beta estimates (linear regression models only), incidence rate ratios (IRR; Poisson regression models only), 95% confidence intervals, and FDR-adjusted *p*-values are reported.

## 3 Results

### 3.1 DTI tractography

Successful tractography was performed on the majority (94%) of WM tracts. The remaining 6.0% of WM tracts could not be isolated due to large tumor volume and distorted anatomy. There was extensive interindividual variability, particularly for streamline number values. The mean FA and streamline number for each WM tract are displayed in **Table 2**. Additional figures displaying the full distribution of FA and streamline number values are presented in the **Supplementary Materials**. Clinical DTI reports noted the following frequencies of WM tract involvement: left AF (25.3%), right AF (20.3%), left cingulum (6.3%), right cingulum (10.1%), left CST (10.1%), right CST (16.5%), left IFOF (21.5%), right IFOF (15.2%), left ILF (12.7%), right ILF (7.6%), left SLF (11.4%), right SLF (10.1%), left uncinate (7.6%), and right uncinate (6.3%). Of note, clinical DTI reports did not comment on smaller tracts (i.e., the fornix and FAT) or specify which subsegments of the SLF or AF were involved.

**Table 2:** Summary of tractography success rate, mean FA, and streamline number for each WM tract.

White matter tract		Number successfully traced (*n* = 79)*^1^*	FA*^1^*	Streamline number*^1^*
*^1^* Mean (SD); *n* (%)				
Anterior AF	Left	73 (92%)	0.505 (0.071)	902.7 (1120.1)
	Right	72 (91%)	0.494 (0.072)	1,209.5 (1704.3)
Cingulum	Left	77 (97%)	0.436 (0.036)	7,024.3 (4297.6)
	Right	79 (100%)	0.421 (0.043)	6,004.0 (3991.4)
Corticospinal tract	Left	78 (99%)	0.556 (0.037)	8,870.5 (3413.4)
	Right	78 (99%)	0.546 (0.039)	7,542.6 (3625.5)
Fornix	Left	66 (84%)	0.400 (0.047)	89.8 (164.1)
	Right	57 (72%)	0.396 (0.036)	102.9 (244.8)
FAT	Left	72 (91%)	0.426 (0.041)	206.5 (479.6)
	Right	72 (91%)	0.424 (0.043)	177.3 (327.9)
IFOF	Left	78 (99%)	0.448 (0.040)	5,042.1 (3228.0)
	Right	78 (99%)	0.458 (0.038)	5,696.2 (3362.1)
ILF	Left	79 (100%)	0.437 (0.042)	5,942.1 (3311.5)
	Right	77 (97%)	0.440 (0.036)	6,484.2 (3685.3)
Long AF	Left	73 (92%)	0.516 (0.075)	1,187.1 (1366.6)
	Right	71 (90%)	0.478 (0.057)	429.5 (976.3)
Posterior AF	Left	79 (100%)	0.446 (0.047)	1,525.4 (1340.8)
	Right	78 (99%)	0.425 (0.043)	871.6 (971.7)
SLF I	Left	73 (92%)	0.556 (0.079)	1,102.2 (1196.3)
	Right	68 (86%)	0.546 (0.083)	1,124.1 (1402.8)
SLF II	Left	73 (92%)	0.490 (0.059)	845.2 (817.0)
	Right	72 (91%)	0.499 (0.053)	1,055.2 (1226.7)
SLF III	Left	79 (100%)	0.474 (0.057)	2,065.1 (1898.1)
	Right	76 (96%)	0.485 (0.059)	2,920.1 (2148.0)
UF	Left	75 (95%)	0.380 (0.039)	675.5 (791.9)
	Right	77 (97%)	0.382 (0.035)	633.1 (726.2)

### 3.2 Neuropsychological results

**Figure 2:**
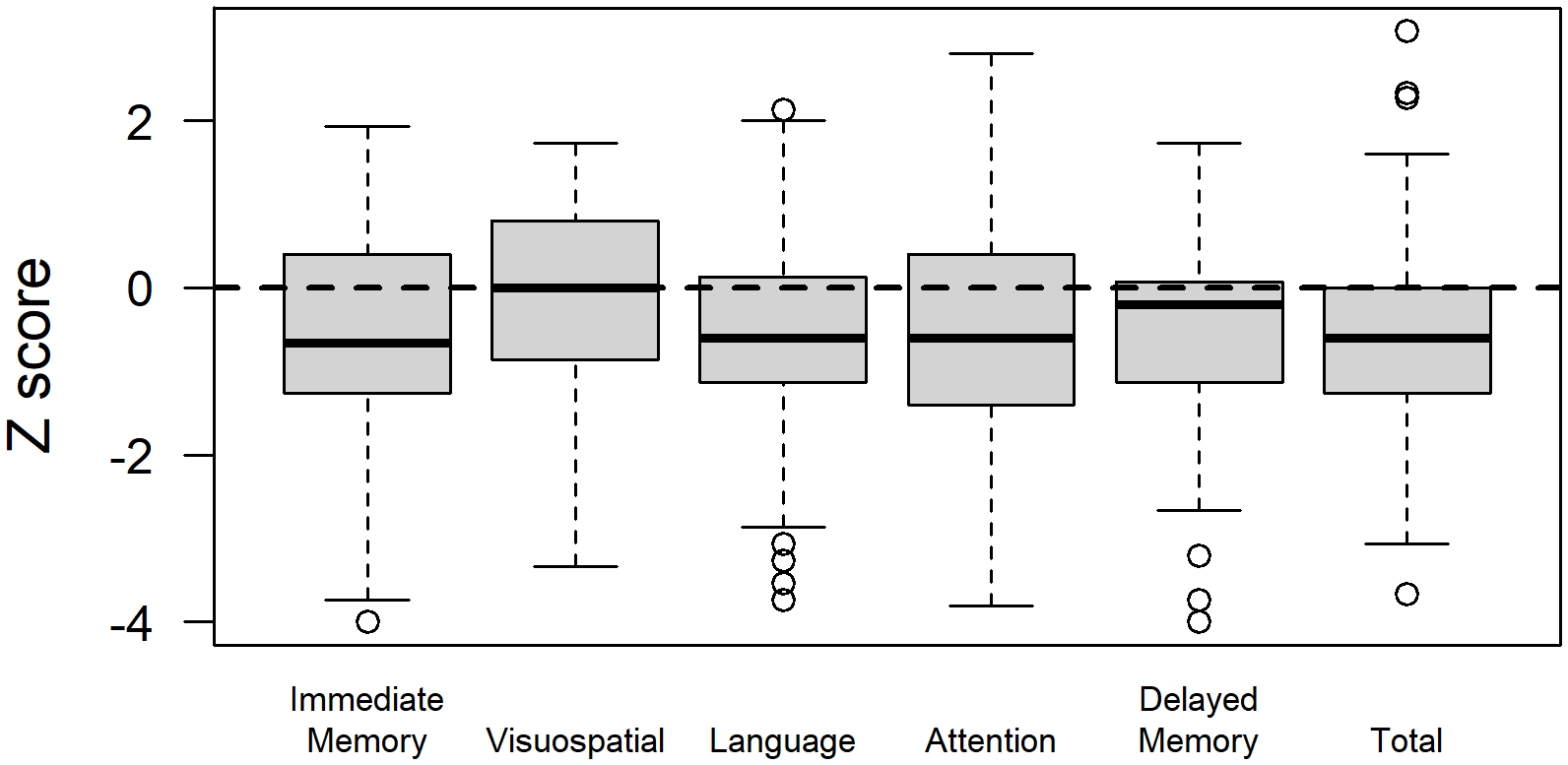
Repeatable Battery for the Assessment of Neuropsychological Status (RBANS) index scores in glioma patients. RBANS index scores are age-corrected and transformed to *z*-scores relative to the normative sample for a total score, as well as scores for the immediate memory, visuospatial, language, attention, and delayed memory domains.

Glioma patients demonstrated variable performance on neuropsychological tests, with a mean total RBANS index *z*-score of −0.5 ± 1.3 compared to the normative sample (**Figure 2**). For individual domains, the mean RBANS index scores trended below those of the general public for immediate memory (*z* = −0.6 ± 1.3), delayed memory (*z* = −0.6 ± 1.2), language (*z* = −0.6 ± 1.2), and attention (*z* = −0.5 ± 1.3). Visuospatial index scores were the least affected in glioma patients (*z* = −0.1 ± 1.2). The complete list of neuropsychological tests is provided in **Table 3**, with descriptions and raw scores. The full distribution of neuropsychological test scores is available in the **Supplementary Materials**.

**Table 3:** Neuropsychological test descriptions and mean scores.

Neuropsychological test	Cognitive abilities tested	Mean raw score (SD)	Total possible points
Memory domain			
RBAS list learning	Immediate verbal recall	25.3 (7.2)	40
RBANS story memory	Immediate verbal recall	17.3 (3.9)	24
RBANS list recognition	Delayed verbal recognition	19.1 (1.4)	20
RBANS list recall	Delayed verbal recall	5.1 (2.7)	10
RBANS story recall	Delayed verbal recall	8.7 (2.7)	12
RBANS figure recall	Delayed visual recall	12.9 (4.5)	20
Language domain			
NAB visual naming	Naming	29.0 (3.3)	31
RBANS visual naming	Naming	9.3 (1.3)	10
RBANs semantic fluency	Semantic processing, working memory	18.7 (6.6)	--
Semantic fluency	Semantic processing, working memory	18.4 (6.7)	--
Phonemic fluency	Phonological processing, working memory	36.6 (15.2)	--
TOPF Word Reading	Reading	47.9 (11.9)	70
Attention domain			
RBANS coding	Visual attention	46.4 (14.5)	89
RBANS digit span total	Attention, auditory recall, working memory	10.2 (2.4)	16
WAIS-IV digit span forward	Attention, auditory recall, working memory	9.9 (2.4)	16
WAIS-IV digit span backward	Attention, auditory recall, working memory	7.9 (2.3)	16
Visuospatial domain			
RBANS figure copy	Visuospatial ability	17.8 (2.8)	20
RBANS line orientation	Visuospatial ability	17.3 (2.9)	20
Executive function domain			
Stroop color–word test	Executive function, cognitive interference	41.6 (15.2)	--
Stroop interference score	Executive function, cognitive interference	2.0 (11.7)	--
Trails A	Visuomotor speed, attention	30.4 (16.9)	--
Trails B	Mental flexibility, attention	81.4 (58.8)	--
WAIS-IV similarities test	Verbal abstract reasoning	25.3 (6.9)	36
Motor			
Grooved pegboard test (DH)	Fine motor dexterity	75.8 (20.2)	--
Grooved pegboard test (NDH)	Fine motor dexterity	87.6 (33.1)	--
Social			
Affect recognition test	Social cognition	17.3 (2.3)	24
Mood			
Beck anxiety inventory	Current mood symptoms	10.0 (10.2)	--
Beck depression inventory	Current mood symptoms	9.6 (8.8)	--

### 3.3 Associations between WM tracts and neuropsychological scores

We found a total of 47 associations between neuropsychological test scores and the WM tract parameters of FA and streamline number. For example, we found correlations between the left cingulum and list learning, the right CST and grooved peg speed (NDH), the left SLF II and phonemic fluency, and the left UF and semantic fluency. The full list of unadjusted findings can be accessed in the **Supplementary Materials**.

**Figure 3:**
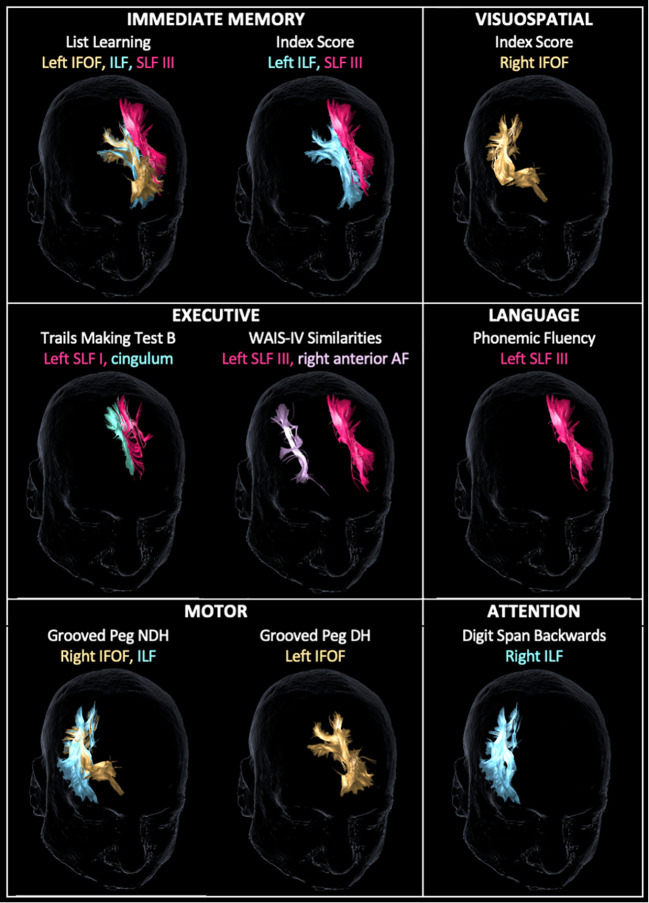
Tractography reconstruction of age-adjusted associations between WM tract integrity (based on fractional anisotropy and/or streamline number) and neuropsychological test scores by domain. Within the immediate memory domain, list learning was predicted by the left inferior frontal-occipital fasciculus (IFOF), inferior longitudinal fasciculus (ILF), and superior longitudinal fasciculus (SLF) III. Immediate memory index score was predicted by the left ILF and SLF III. Visuospatial index score was predicted by the right IFOF. Within the executive domain, Trail Making Test B score was predicted by the left SLF I and cingulum, and Wechsler Adult Intelligence Scale – Fourth Edition (WAIS-IV) Similarities score was predicted by the left SLF III and right anterior arcuate fasciculus (AF). Within language, phonemic fluency was predicted by SLF III. Within the motor domain, grooved peg speed of the non-dominant hand (NDH) was predicted by the right IFOF and ILF, and grooved peg speed of the dominant hand (DH) was predicted by the left IFOF. Within attention, backward digit span score was predicted by the right ILF. WM tracts are displayed in the same color as their corresponding labels.

Following a more conservative adjustment for age to account for the linear decrease of FA values and neuropsychological outcomes over time (32), there were 16 significant associations between WM tracts and neuropsychological scores (**Figure 3**). The left IFOF predicted list learning by FA (*β* = 65.7, *p* < 0.023) and streamline number (*β* = 0.0008, *p* = 0.029) and DH grooved peg speed by streamline number (*β* = −0.0023, *p* = 0.008). The right IFOF FA was associated with NDH grooved peg speed (*β* = −312.9, *p* = 0.048) and RBANS visuospatial index score (irr = 12.7, *p* = 0.021). The left ILF FA predicted list learning (*β* = 66.5, *p* = 0.031) and immediate memory index scores (*β* = 11.9, *p* = 0.03). The right ILF predicted NDH grooved peg speed by FA (*β* = −369.8, *p* = 0.014) and backward digit span score by streamline number (*β* = 0.0002, *p* = 0.023). The left SLF I FA was associated with processing speed on the Trail Making Test Part B (*β* = −2.4, *p* = 0.032). The left SLF III FA was associated with list learning (*β* = 50.4, *p* = 0.006), immediate memory index score (*β* = 9.1, *p* = 0.015), phonemic fluency (*β* = 106.0, *p* = 0.012), and verbal abstract reasoning on the WAIS-IV Similarities test (irr = 9.8, *p* < 0.001). The left cingulum streamline number was associated with processing speed on the Trail Making Test Part B (*β* = −0.0001, *p* = 0.023). The right anterior AF streamline number was associated with verbal abstract reasoning (irr = 0.99, *p* = 0.041). The CST, FAT, UF, and fornix did not demonstrate age-adjusted associations with neuropsychological tests in our dataset.

## 4 Discussion

In this study, we provide novel evidence that the disruption of individual WM tracts is associated with specific preoperative cognitive deficits in patients with gliomas, adding to the current understanding of the functional organization of the brain. Our data demonstrated that large association tracts like the IFOF, ILF, and SLF may contribute to multiple complex cognitive functions. Specifically, we found that the left IFOF predicts immediate memory and fine motor dexterity; the right IFOF predicts fine motor dexterity and visuospatial ability; the left ILF predicts immediate memory; the right ILF predicts fine motor dexterity and attention; the left SLF I predicts processing speed; the left SLF III predicts immediate memory, phonemic fluency, and verbal abstract reasoning; the left cingulum predicts processing speed; and the right anterior AF predicts verbal abstract reasoning. Given the neuroplastic potential of glioma patients (13, 16), possible exclusion of patients with the most severe cognitive deficits due to inability to perform tractography, and statistical adjustment for age, the relationships we report are quite stringent and, as such, likely represent critical functional correlates, but do not rule out the importance of other tracts. These data highlight critical WM association fibers to assess during presurgical planning and intraoperative stimulation to maximize functional preservation in glioma patients.

### 4.1 DTI measures of WM tracts

Tractography was successfully performed for the majority of tracts and there was variable integrity of each tract, as shown by the range of FA and streamline number values (**Table 2**, **Supplementary Materials**). This variability likely reflects pathological changes induced by glioma as well as baseline variations in the anatomical structure and function of the brain (4). Overall, there were more significant associations found between neuropsychological test scores and FA values than between test scores and streamline number values. This may suggest that loss of WM tract integrity in glioma patients is more related to axonal disorganization and increased cellular water, as represented by FA, rather than direct axonal damage or degeneration, as represented by streamline number (4, 6, 12). For instance, cognitive deficits induced by brain tumors are often related to edema or compression of adjacent normal structures, with resection providing resolution of these deficits (34). Additionally, quantification of streamline number with manual ROIs may be less reliable in regions of significant streamline displacement. Nonetheless, by combining FA and streamline number data, we may detect more subtle associations between structural integrity and cognitive function.

### 4.2 Neuropsychological deficits

Glioma patients can exhibit preoperative impairments in every cognitive domain and 62.6% have an impairment in at least one domain (7). Consistent with previous studies (8, 35), we found that glioma patients demonstrated relatively mild cognitive deficits across the language, attention and memory, and executive domains, and the visuospatial domain was the least clinically affected. Previous studies have suggested that executive function may be particularly vulnerable to effects of a glioma because it relies on coordinated neurocognitive processes from widespread brain networks (8). On the other hand, some authors hypothesize that visuospatial performance may be preserved in glioma patients due to less constrained redistribution of neural information between the widespread neural connections to the occipital lobe, including the IFOF, the ILF, and other WM tracts (13). In our patient cohort, there were fewer gliomas in a posterior lobe location, which has been found to be correlated with visuospatial function; this likely contributed to the presence of fewer deficits in this domain (8).

### 4.3 WM associations with cognitive function

The IFOF is the longest association tract in the human brain, with extensive terminations in the frontal, temporal, parietal, and occipital lobes (24). Previous studies using DES have identified diverse functional correlates of the IFOF, primarily involving semantic processing as the “ventral pathway” within Hickok and Poeppel’s dual-stream model of language (36–38). Other authors have demonstrated a role of the IFOF in visual recognition and goal-directed behavior, likely mediated by its anatomic terminations to the occipital lobe and frontoparietal executive network, respectively (39). We similarly found that the left and right IFOF predict contralateral fine motor dexterity and the right IFOF predicts visuospatial ability. These findings may emphasize the clinical significance of motor abilities relying on multiple intact domains, such as visual and executive functioning. Consistent with previous data in glioma patients, we also detected an association between the left IFOF and verbal memory (16). Overall, the IFOF may play an essential role in multiple cognitive functions due to its large size and extensive connectivity, particularly including functions that rely on visual or semantic processes.

The ILF is a long-range association tract that connects the occipital and anterior temporal lobes (40). It is involved in the transfer of information between the visual, memory, and limbic territories (36). Hence, it contributes to a wide range of cognitive functions including object recognition, memory, reading, lexical and semantic processing, and emotion processing (34, 36, 40). Some of these processes are considered to be relatively lateralized, such as the right ILF contributing to visual functions and the left ILF contributing to lexical processes, but DES studies in glioma patients have suggested the potential for significant bilateral ILF functional compensation (40). We correspondingly found that the left ILF predicts list learning and the right ILF predicts fine motor dexterity on the grooved pegboard test, which also may reflect its critical role in visually guided tasks and object recognition (40). We also detected an association between the right ILF and attention on backward digit span, a task that may require more bilateral neural recruitment to manipulate the sequence of presented information (41). Taken as a whole, our findings provide supportive evidence that the ILF subserves several complex cognitive functions in glioma patients.

The SLF is considered to be the largest association bundle in the human brain and is composed of three segments that connect widespread regions of the frontal and parietal lobe (42). The left SLF, along with the AF, is thought to mediate phonological processing as the dorsal stream of language (38). Nonetheless, recent studies suggest broader roles of the SLF in higher cognition, including metacognition (i.e., reflecting on one’s own cognitive processes) (43) and depressive rumination (44), which require intact attention and executive control, among other processes. We found associations between the left SLF III and phonemic fluency, immediate memory, and verbal abstract reasoning and between the left SLF I and executive functioning on the Trail Making Test Part B, which assesses mental flexibility and attention. Therefore, our data support a broader role of the SLF in language as well as verbal, executive, and memory tasks. Given the left-sided lateralization of our findings, further studies will be necessary to delineate whether the deficits noted on memory and executive tasks result primarily from underlying impairments in language production or whether the SLF contributes separately to these processes.

The AF is a left-lateralized association tract with three subcomponents that connects the perisylvian language areas of the frontal, parietal, and temporal lobes. Like the SLF, the left AF has a well-established role in language, particularly in phonemic processing (16, 38). On the other hand, the role of the right AF is less clear, but alterations in this tract have been proposed as markers of impaired social cognition (45) and spatial attention (46). Interestingly, Catani et al. demonstrated that individuals with more symmetric distributions of the left and right AF perform better at learning and remembering words based on semantic association (28). We similarly found that right anterior AF integrity predicts verbal abstract reasoning on the WAIS-IV Similarities Test. Therefore, we hypothesize that greater right AF connectivity may be protective to some extent against semantic impairments in glioma patients.

The cingulum connects medial regions of the frontal, parietal, and temporal lobes. The cingulum is most frequently found to be correlated with executive functions (4) and also plays proposed roles in emotional processing as a component of the limbic system, as well as connecting regions of the default mode network (DMN), which largely mediates cognitive and social processing at rest (47, 48). Consistent with previous studies, we found that the left cingulum predicts executive functioning on the Trail Making Test Part B, providing additional evidence that the cingulum plays an important role in higher-level cognitive functioning.

Before adjusting for age, we identified additional associations that have been established by previous studies, including associations between the right SLF III and spatial attention (49), the CST and motor ability (50), and the UF and semantic fluency (38). However, after age adjustment, we did not detect these expected associations or other functional correlates of the UF, fornix, FAT, and CST. It is possible that the gliomas in our patient population did not evenly affect all WM tracts, particularly smaller tracts like the FAT and fornix that do not provide large, longitudinal connections to multiple ipsilateral lobes of the brain. For instance, on clinical DTI reports, radiologists commented relatively less frequently on involvement of the UF, which may have yielded fewer detectable functional correlates of this tract. Other correlations may have been difficult to detect due to neuroplasticity, high interindividual structural variability, compensation on tasks with other cognitive abilities, or low patient numbers inherent to our study.

### 4.4 Limitations

There are several limitations to this study. DTI may have suboptimal performance in regions of crossing or merging fibers, particularly in the setting of lesion-induced edema, tissue compression, and degeneration (10, 51). During tractography, a tumor may alter the anatomical landmarks for ROI creation or displace the typical pathway by which the fibers connect (25, 51). Quantifying the number of streamlines is especially subject to this possibility of error. Additionally, excluding the FA values of tracts that could not be reconstructed in regions of significant anatomical disruption may have produced a more conservative estimate of tract associations. We defined ROIs manually to overcome some challenges of anatomic variation (24). However, manual ROI placement is also subject to variability, which we aimed to minimize by selecting one researcher to perform tractography using systematically placed ROIs based on previously published approaches (2, 24). Overall, we are limited in making broader conclusions about neurological changes seen in glioma patients due to the lack of a control group without gliomas. Despite this, our dataset contains WM tracts in the hemisphere contralateral to each tumor, which may serve as a reference point for relatively preserved WM tracts. Future studies with postoperative longitudinal data will be important to further characterize specific WM alterations caused by tumor and treatment, and to assess the corresponding changes in cognition.

## 5 Conclusions

Overall, our findings support the idea that WM tracts play an essential role in mediating complex cognitive functions. Large association tracts connecting widespread regions of the brain like the ILF, IFOF, and SLF III may contribute to multiple functions. DTI tractography should be included in presurgical planning and subcortical intraoperative mapping, in conjunction with these findings, to help optimize maximum tumor resection and cognitive preservation, especially for complex cognitive functions that may be difficult to test intraoperatively. Our findings also add to the growing body of literature indicating that different patterns of WM injury may provide important prognostic information about symptom presentation and disease progression (4). This knowledge may inform personalized approaches to therapy and recovery for patients with glioma.

## Data availability statement

The raw data supporting the conclusions of this article will be made available by the authors, without undue reservation.

## Ethics statement

The studies involving humans were approved by Northwestern University Institutional Review Board. The studies were conducted in accordance with the local legislation and institutional requirements. The participants provided their written informed consent to participate in this study.

## Author contributions

MA contributed to the study design, tractography, the first draft of the manuscript, and final review. M-AM contributed to the study design, collected neuropsychological data, and reviewed and edited the manuscript. DA performed statistical analysis and contributed to the first draft and revision of the manuscript. MT designed the study and revised the manuscript. All authors contributed to the article and approved the submitted version.
