# Supplementary material for: White matter tracts contribute selectively to cognitive functioning in patients with glioma
Source: Front Oncol. 2023 Oct 20;13:1221753. doi: 10.3389/fonc.2023.1221753 (PMC10623310; doi:10.3389/fonc.2023.1221753)

eFigure 1A. Histograms and Boxplots of Fractional Anisotropy (FA) Values

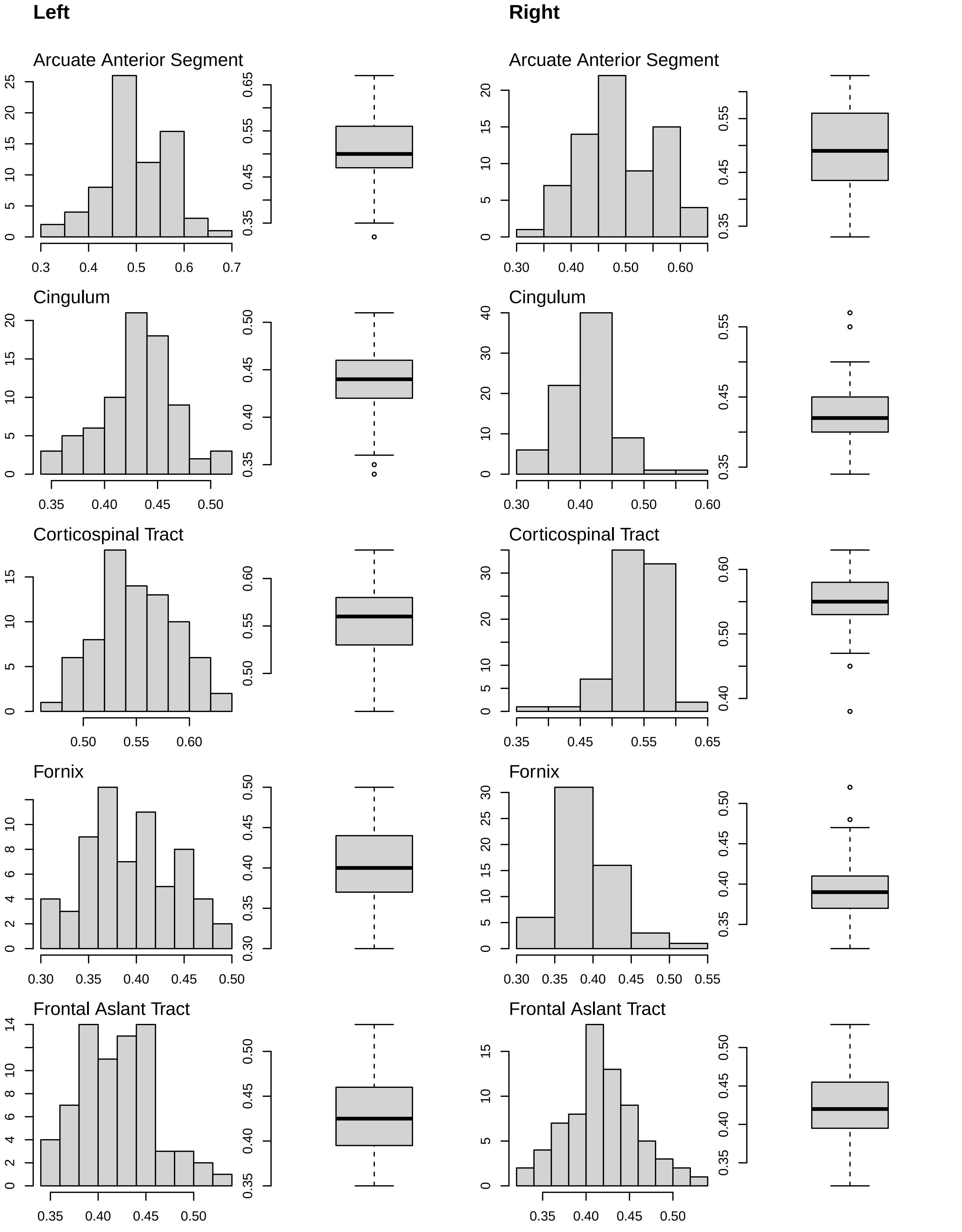

eFigure 1B. Histograms and Boxplots of Fractional Anisotropy (FA) Values

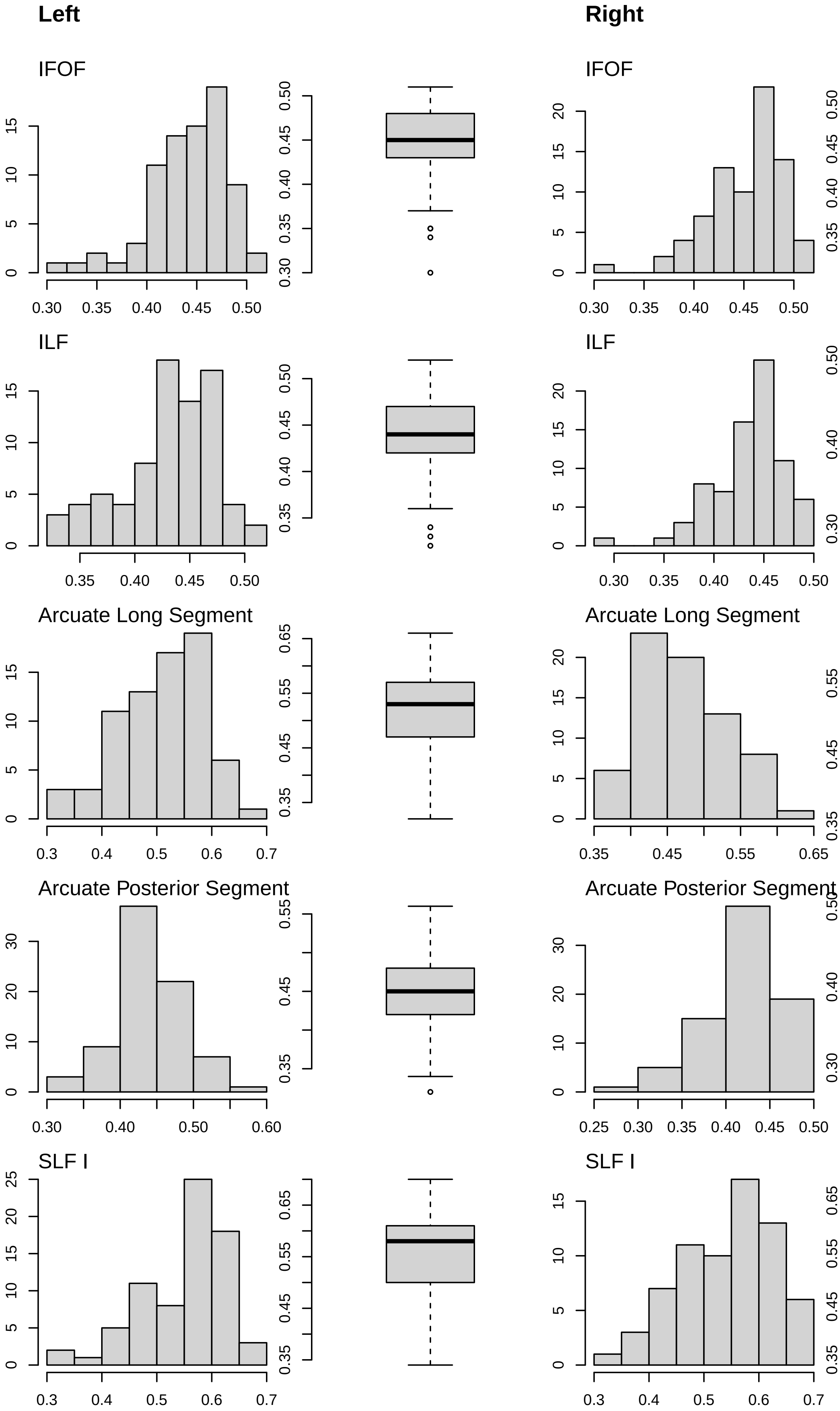

eFigure 1C. Histograms and Boxplots of Fractional Anisotropy (FA) Values

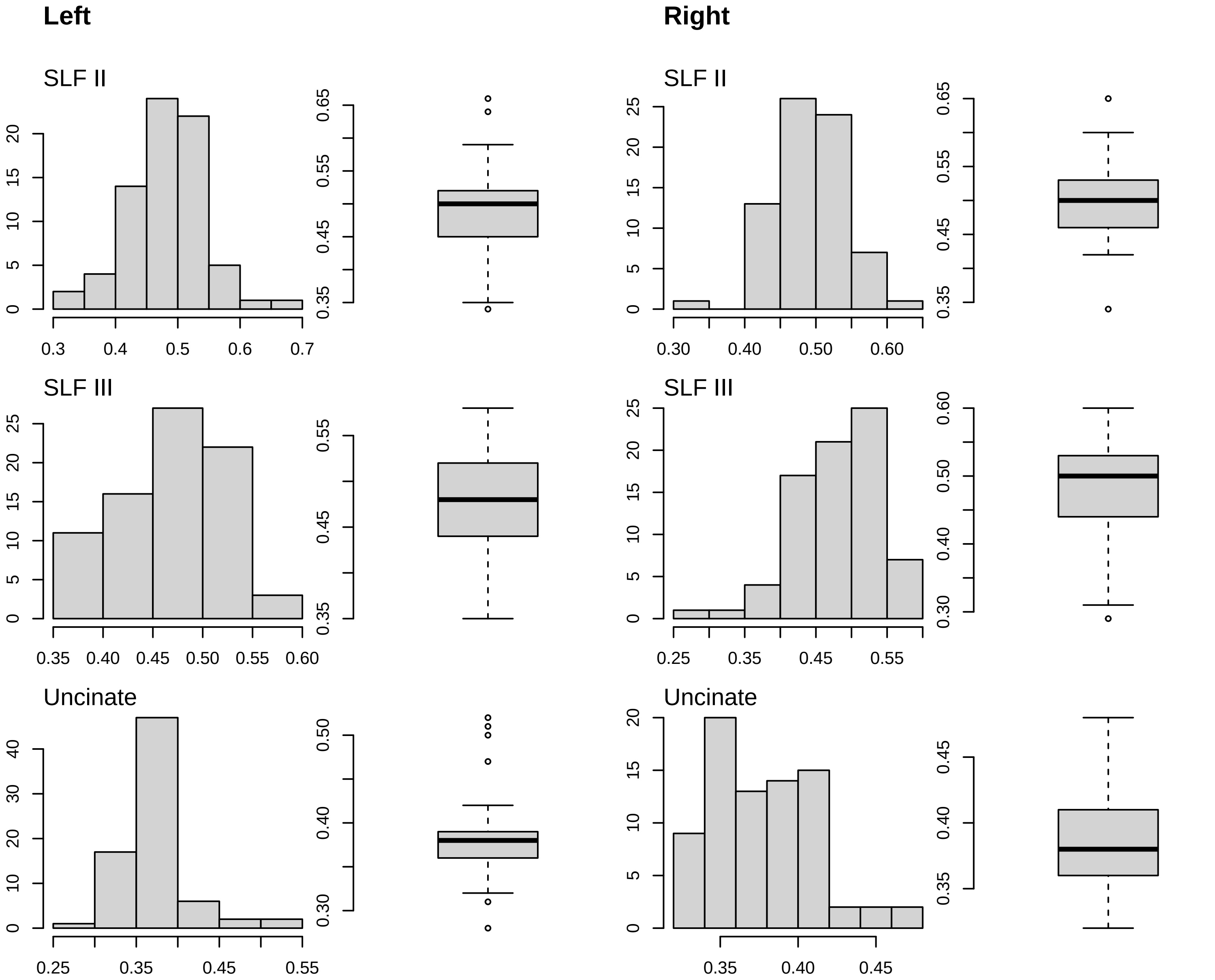

Supplement: Supplementary file 2 [file DataSheet_1.pdf]
