# Supplementary material for: White matter tracts contribute selectively to cognitive functioning in patients with glioma
Source: Front Oncol. 2023 Oct 20;13:1221753. doi: 10.3389/fonc.2023.1221753 (PMC10623310; doi:10.3389/fonc.2023.1221753)

eFigure 2A. Histograms and Boxplots of Streamline Number Values

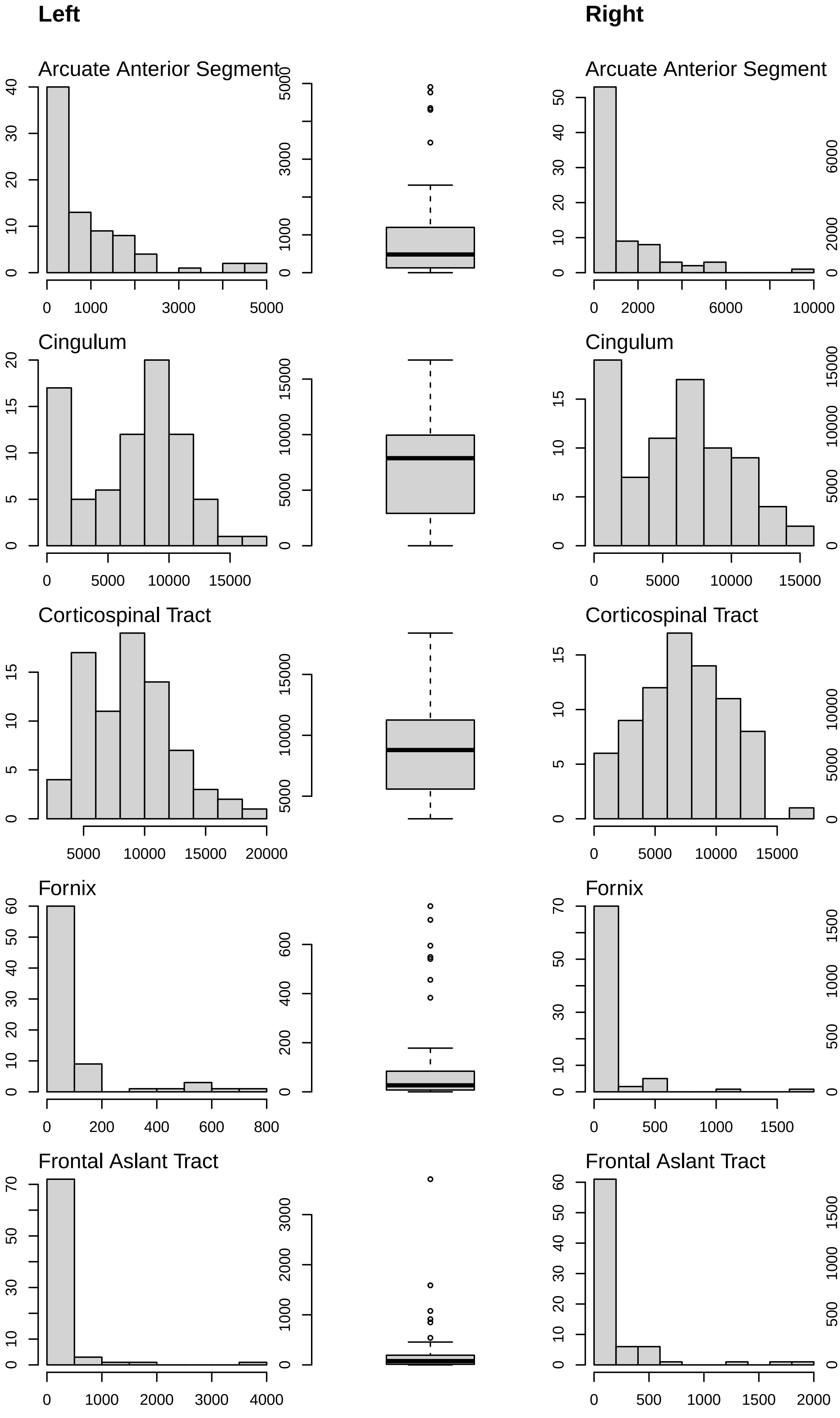

eFigure 2B. Histograms and Boxplots of Streamline Number Values

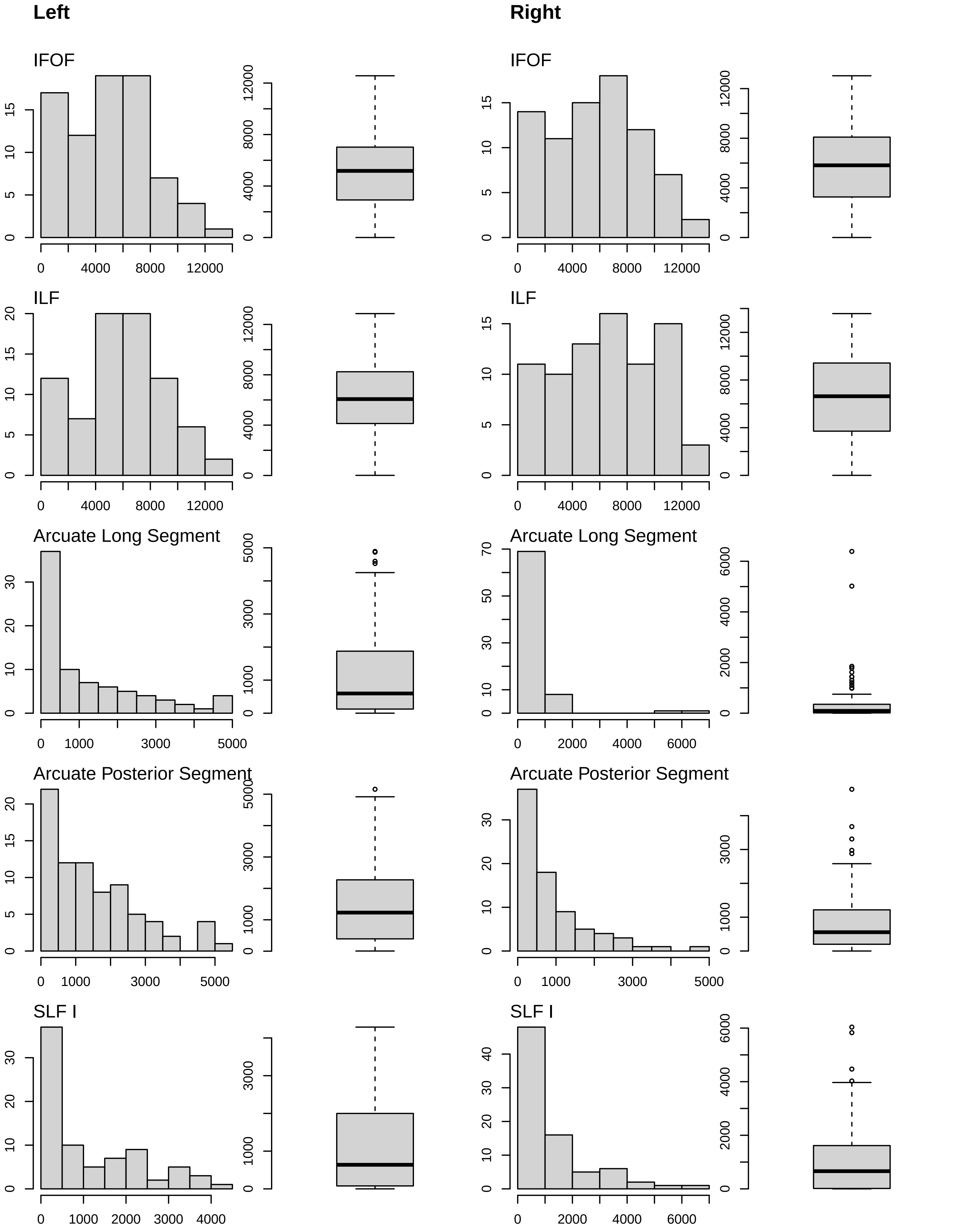

eFigure 2C. Histograms and Boxplots of Streamline Number Values

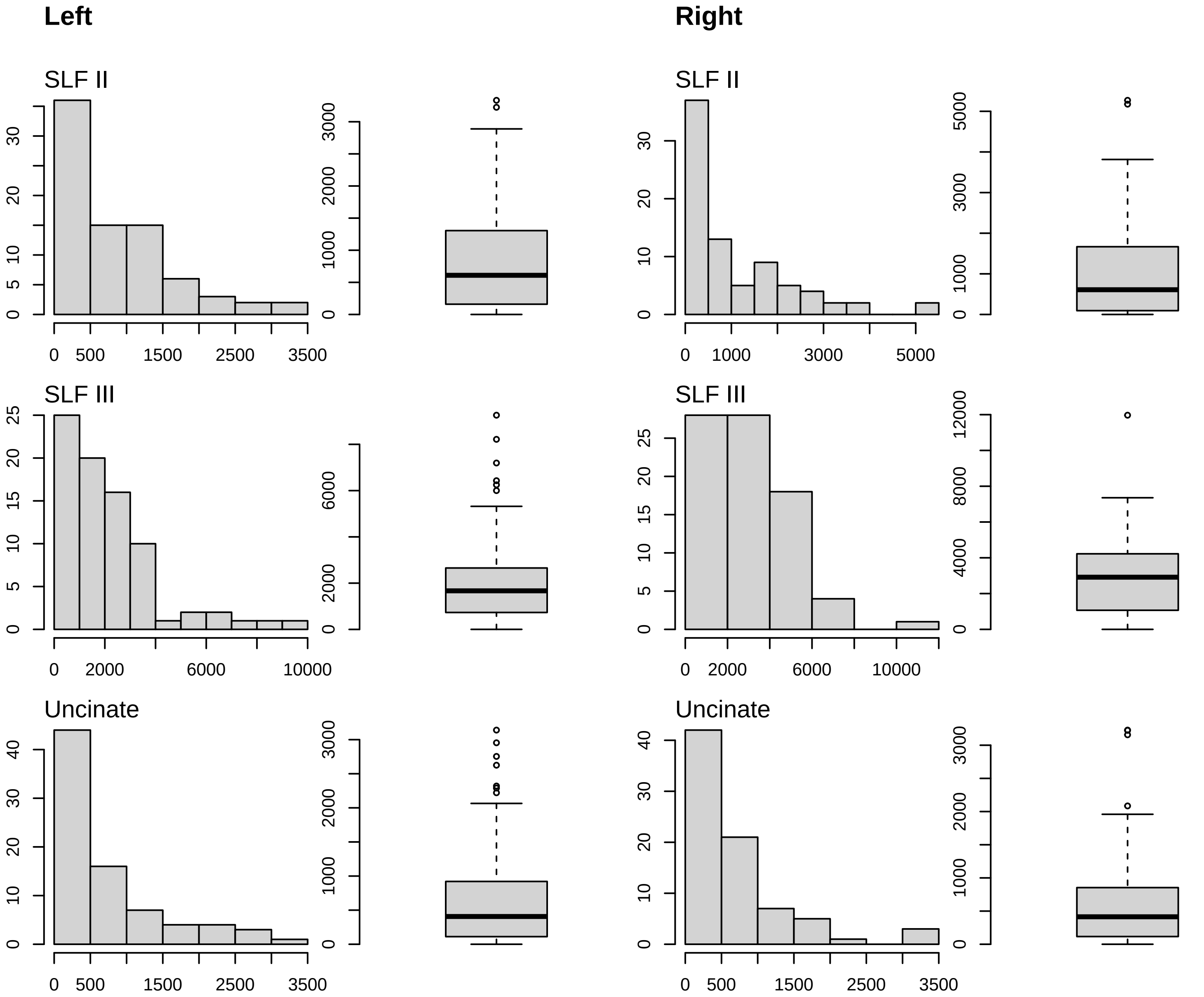

Supplement: Supplementary file 3 [file DataSheet_2.pdf]
