# Supplementary material for: White matter tracts contribute selectively to cognitive functioning in patients with glioma
Source: Front Oncol. 2023 Oct 20;13:1221753. doi: 10.3389/fonc.2023.1221753 (PMC10623310; doi:10.3389/fonc.2023.1221753)

eFigure 3A. Histograms and Boxplots of Neuropsychological Test Values

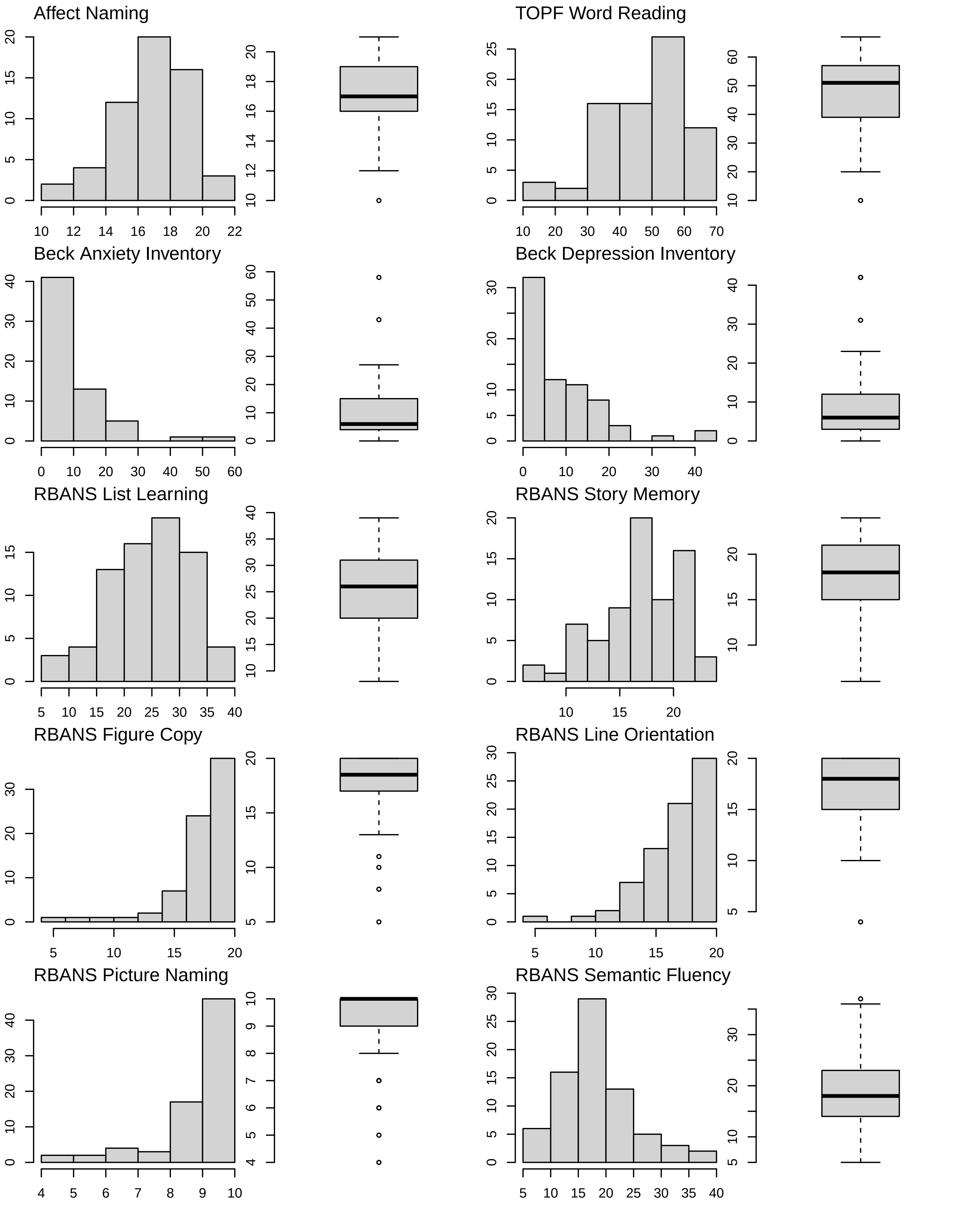

eFigure 3B. Histograms and Boxplots of Neuropsychological Test Values

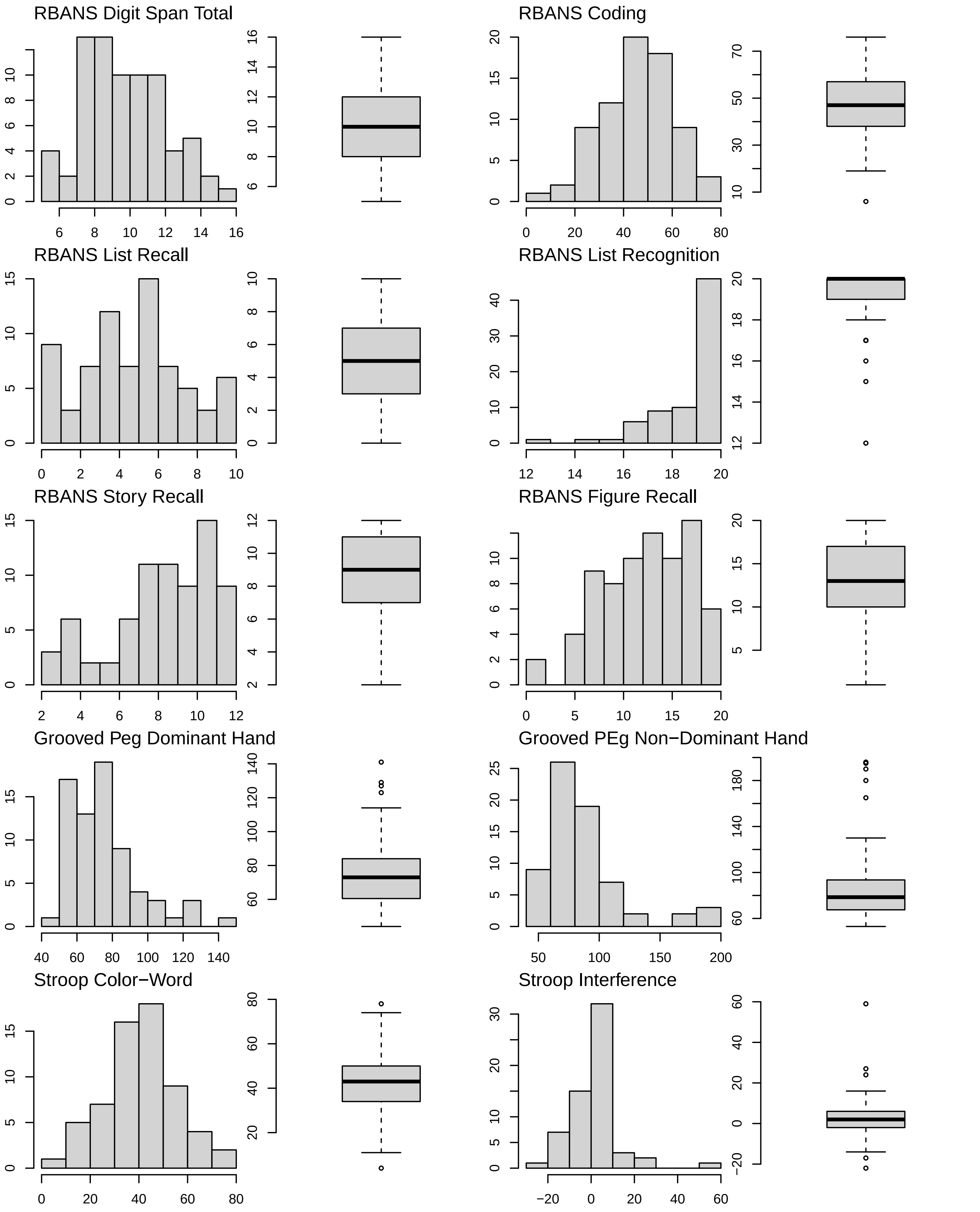

eFigure 3C. Histograms and Boxplots of Neuropsychological Test Values

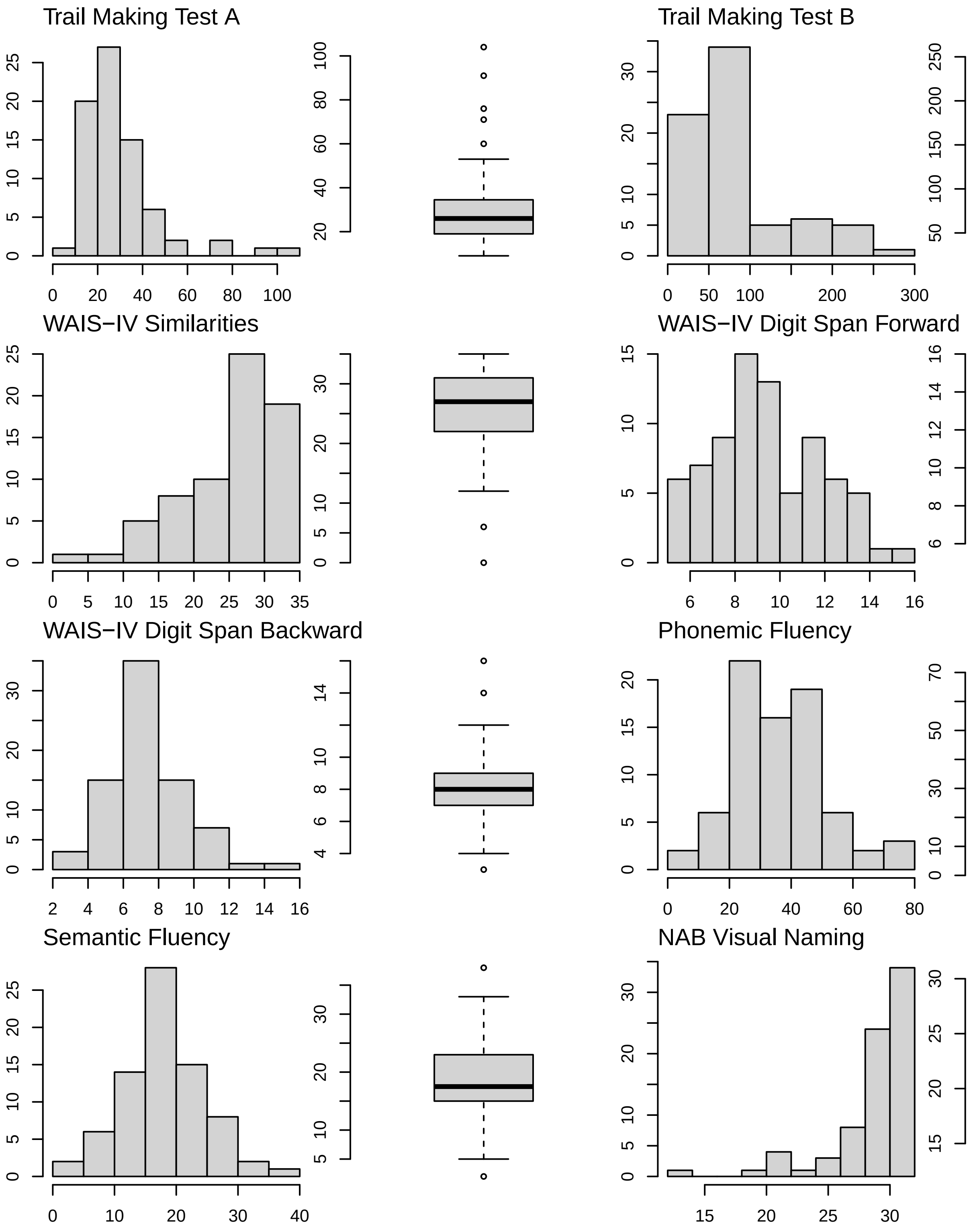

Supplement: Supplementary file 4 [file DataSheet_3.pdf]
